# Impact of frailty on inpatient outcomes in thyroid cancer surgery: 10-year results from the U.S. national inpatient sample

**DOI:** 10.1186/s40463-020-00450-5

**Published:** 2020-07-22

**Authors:** Dong Xu, Mengjia Fei, Yi Lai, Yuling Shen, Jiaqing Zhou

**Affiliations:** Department of Head and Neck Surgery, Renji Hospital, School of Medicine, Shanghai Jiao Tong University, No. 145 Middle Shandong Road, Shanghai, 200001 China

**Keywords:** Frailty, National inpatient sample (NIS), Thyroid cancer, Thyroidectomy

## Abstract

**Background:**

Frailty is linked to perioperative morbidity and mortality. We evaluated the impact of preoperative frailty on inpatient outcomes of patients undergoing surgery for thyroid malignancy.

**Methods:**

This population-based, retrospective observational study extracted data of hospitalized patients who were 18 years and older with a primary diagnosis of thyroid cancer undergoing thyroidectomy from the US Nationwide Inpatient Sample (NIS) database (2005–2014). Participants were stratified into frail and non-frail using the Johns Hopkins (ACG) frailty-defining diagnosis indicator. Study endpoints were in-hospital mortality, incidence of surgical and medical complications and prolonged length of stay. Univariate and multivariate analysis were performed to determine associations between the endpoints and frailty.

**Results:**

Data of 38,202 patients were included. After adjusting for possible confounders, frailty remained significantly associated with higher odds of in-hospital mortality (OR: 3.839, 95% CI: 1.738–8.480), prolonged length of stay (OR: 5.420, 95% CI: 3.799–7.733), surgical complications (OR: 3.144, 95% CI: 2.443–4.045) and medical complications (OR: 6.734, 95% CI: 5.099–8.893) compared with non-frailty. In patients > age 65 years, adjusted odds ratio for frailty was 4.099 (95% CI: 1.736–9.679) for in-hospital mortality, 6.164 (95% CI: 3.514–10.812) for prolonged length of stay, 3.736 (95% CI: 2.620–5.328) for surgical complications, and 5.970, 95% CI: 4.088–8.720 for medical complications, all with significance.

**Conclusion:**

Frailty is associated with increased risk for adverse inpatient outcomes, including prolonged hospital stay, surgical and medical complications and mortality independent of age and comorbidities in thyroid cancer patients undergoing surgery. Study findings may provide valuable information for preoperative risk stratification.

## Introduction

Frailty is highly prevalent among older adults worldwide and is associated with increased risk of falling and hospitalizations, high disability, morbidity and mortality [1, 2]. Frailty is defined as a syndrome of lower energy reserve and increased vulnerability to stressors during aging along with age-associated declines in lean body mass, strength, endurance and activity levels [1]. Frailty has also been linked to perioperative morbidity and mortality among older adults in various surgical arenas, including vascular surgery [3], cardiac [4] and elective non-cardiac surgeries [5]. Frailty is shown to be more significant than age itself in predicting poor outcomes [2, 6]. As such, frailty is a recognized factor in geriatric oncology, with increased risk of adverse outcomes in older adult cancer patients [6, 7]. More than half of older adult cancer patients were found to have either pre-frailty or frailty, increasing risk of chemotherapy intolerance, postoperative complications and 30-day mortality [7].

Thyroid cancer is the most common malignancy in endocrine system organs and its incidence is increasing globally. In the United States, the annual percentage change was 2.4% between the 1980s to 1997 and increased to 6.6% between 1997 and 2009 [8]. Many researchers attribute this increase to enhanced detection through more sensitive screening and diagnostic procedures [9, 10]. In fact, the increased incidence in the U.S. is considered to be of epidemic proportions, especially in women, who have higher thyroid cancer rates but lower prevalence at autopsy than men—a trend suggesting that the epidemic is one of higher detection rates than actual disease rates [10]. Although thyroid cancer is more commonly diagnosed at younger ages, and is associated with a good overall prognosis, older adults with thyroid cancer are challenged by a more aggressive disease that can lead to significant morbidity as well as to a significant economic burden on healthcare systems [11–13]. In particular, older adults undergoing thyroidectomy for thyroid cancer are prone to more complications, greater risk of recurrence and distant metastases and reduced overall and disease-free survival [11].

Prior studies have investigated the effects of frailty on patients undergoing surgery for a broad range of thyroid disease [14–16]. Frailty was more predictive of post-thyroidectomy systemic complications than age in older adults with multimodal goiter [14]. In common ambulatory general surgery operations including thyroid surgeries, frailty was associated with increased perioperative morbidity independent of age [15]. A preoperative risk index based on multidimensional framework also incorporated frailty as a major component and was found acceptable to determine major adverse events after thyroid or parathyroid surgery, whereas not for malignancy specifically [16].

However, despite the evidence gained from these studies, the impact of frailty on the outcomes of older adults undergoing surgery for thyroid malignancy has yet to be investigated directly. Therefore, the present study aimed to evaluate the impact of preoperative frailty on inpatient outcomes in older adults undergoing surgery for thyroid malignancy.

## Methods

### Study design and data source

This population-based, retrospective observational study extracted all data from the US Nationwide Inpatient Sample (NIS) database, which is the largest all-payer, continuous inpatient care database in the United States, including about 8 million hospital stays each year [17]. It is administered by the Healthcare Cost and Utilization Project (HCUP) of the National Institutes of Health (NIH). Patient data include primary and secondary diagnoses, primary and secondary procedures, admission and discharge status, patient demographics, expected payment source, duration of hospital stay, and hospital characteristics (i.e., bed size/location/teaching status/hospital region). All patients are initially considered for inclusion. The NIS database derives patient data from about 1050 hospitals from 44 States in the US, sampled to represent a 20% stratified sample of US community hospitals as defined by the American Hospital Association.

### Ethics statement

All data were obtained through request to the Online Healthcare Cost and Utilization Project (HCUP) Central Distributor (available at: https://www.distributor.hcup-us.ahrq.gov/), which administers the database (certificate # HCUP-4R08J60JV). This study conforms to the data-use agreement of the NIS from HCUP. As this study was an analysis of secondary data from the NIS database, patients and the public were not involved directly. The study protocol was submitted to the Institutional Review Board (IRB) of Renji Hospital, School of Medicine, Shanghai Jiao Tong University, which exempted the study from IRB approval. Since all data in the NIS database are de-identified, the requirement for informed consent was also waived.

### Study population

Adults ≥18 years old admitted to U.S. hospitals between 2005 and 2014 with a primary diagnosis of thyroid cancer undergoing thyroidectomy were identified in the NIS database through the International Classification of Diseases, Ninth Revision (ICD-9) diagnostic codes (code 193) and procedure codes (code 06.2, 06.3, 06.4, 06.5). Participants with no information on mortality status or length of stay were excluded.

The participants were further stratified into frail and non-frail groups, based on the 10 clusters of frailty-defining diagnoses that comprise the Johns Hopkins Adjusted Clinical Groups (ACG)frailty-defining diagnosis indicator, a binary variable, using ICD-9 codes assigned during admission, as described previously [18, 19]. Details of relevant codes for frailty are shown in Table 1.

**Table 1:** ICD-9 codes for defining frailty and complications

Variable	Diagnoses	ICD-9
Frailty		
Malnutrition	Nutritional marasmus Other severe protein-calorie malnutrition	261, 262, 263.8, 263.9, V77.2,
Dementia	Senile dementia with delusional or depressive features Senile dementia with delirium	290.20, 290.21, 290.3
Severe vision impairment	Profound impairment, both eyes Moderate or severe impairment, better eye/lesser eye: profound	369.0, 369.00, 369.01, 369.03, 369.04, 369.06, 369.07, 369.08,
Decubitus ulcer	Decubitus ulcer	707.0, 707.00, 707.01, 707.02, 707.03, 707.04, 707.05, 707.06, 707.07, 707.09, 707.20, 707.21, 707.22, 707.23, 707.24, 707.25
Urinary incontinence	Incontinence without sensory awareness Continuous leakage	788.34, 788.37
Loss of weight	Abnormal loss of weight and underweight Feeding difficulties and mismanagement	783.2, 783.21, 783.22, 783.3
Fecal incontinence	Fecal incontinence	787.6
Social support needs	Lack of housing Inadequate housing Inadequate material resources	V60.0, V60.1 V60.2
Difficulty in walking	Difficulty in walking Abnormality of gait	719.7, 781.2
Fall	Fall on stairs or steps Fall from wheelchair	E880, E880.0, E880.1, E880.9, E884.3
**Surgical complications**		
Tracheostomy		519.0, 519.00, 519.01, 19.02, 519.09
Hoarseness		784.4, 784.40, 784.41, 84.42, 784.43, 784.44, 784.49, 784.51
Hemorrhage, hematoma, or seroma		285.1, 998.1, 998.11, 998.12, 998.13
Cystitis		595, 595.0, 595.3, 595.4, 595.8, 595.89, 595.9
Hypocalcemia		275.41, 275.49
Vocal cord paresis or paralysis		478.3, 478.30, 478.31, 478.32, 478.33, 478.34
Wound complications		998.3, 998.30, 998.31, 998.32, 998.33, 998.83
Technical complications		998.2, 998.4, 998.5, 998.51, 998.59, 998.6, 998.7, 998.8, 998.81, 998.89, 998.9
**Medical complications**		
Shock		998.0
Cardiovascular		410.0–410.9, 411.1, 411.8, 415.0, 420.0, 420.9, 421.0, 421.1, 421.9, 422.0, 422.9, 427.0–427.5, 428.0–428.9, 451.11, 997.00, 997.01, 997.02, 997.09, 997.2, 997.79
Pulmonary		512.1, 518.4, 518.81, 518.82, 518.84, 997.3, 997.31, 97.32, 997.39
Acute kidney injury		584.5–584.9
Pneumonia		480,480.0, 480.1, 480.2, 480.3, 480.8, 480.9, 481, 482, 482.0, 482.1, 482.3, 482.30, 482.31, 482.32, 482.39, 482.40, 482.41, 482.42, 482.49, 482.8, 482.81, 482,82, 482.83, 482.84, 482.89, 482.9, 483, 483.1, 483.8, 484, 484.1, 484.3, 484.5, 484.6, 484.7, 484.8, 485, 487.0, V12.61, 507.0, 514, 518.4, 518.5, 516, 516.8, 997.31
Infection / Sepsis		038.0–038.9, 519.2, 785.52, 995.9, 996.31, 996.62, 996.64, 999.3, 998.5, 998.51, 998.59, 599.0, V13.02

### Study variables and outcome measures

Study endpoints were in-hospital mortality, incidence of any surgical complications, any medical complications and prolonged length of stay. Surgical complications included tracheostomy, hoarseness, hemorrhage, hematoma or seroma, cystitis, hypocalcemia, vocal cord paresis or paralysis, wound and technical complications. Medical complications included shock, cardiovascular, pulmonary, acute kidney injury, pneumonia, infection and sepsis. The details of relevant codes are summarized in Table 1. Prolonged length of stay was defined as length of stay ≥75th percentile of the study cohort.

#### Covariates

Patients’ characteristics included age (grouped into: < 45, 45–64, and ≥ 65 years), gender, race/ethnicity, household income level, insurance status (primary payer), and admission type (elective or emergent). Procedure types were divided into: unilateral / partial thyroidectomy, total (complete) thyroidectomy, and substernal thyroidectomy. Patients requiring cervical lymph node dissection, or with tumor metastasis to the lung or bone, smoking status, obesity or overweight status were also identified through ICD-9 diagnostic or procedure codes. Comorbidities were graded using the Romano adaptation of the Charlson comorbidity index [20, 21], excluding codes for the index cancer diagnosis from the solid tumor category, in accordance with previous study [22]. Hospital-related characteristics (bed size/location/teaching status/hospital region) and hospital volume of thyroidectomy cases were also extracted from the database as part of the comprehensive data available for all participants.

### Statistical analysis

All categorical variables are expressed as counts (percentages). Comparisons of proportions between groups for categorical variables were performed using Pearson’s chi-square test or Fisher’s exact test. Univariate and multivariate analysis were performed to determine the associations between frailty and in-hospital mortality, prolonged length of stay (of survivors), incidence of surgical complications, and incidence of medical complications. Additional stratified analyses were performed according to age group and number of comorbidities. All statistical analyses were performed using SAS statistical software version 9.4 (SAS, Cary, NC, USA). Two-sided *p*-values less than 0.05 were considered statistically significant.

## Results

Data of 38,202 patients aged 18 years or older who were diagnosed with thyroid cancer and undergoing thyroidectomy were extracted from the NIS database (2005–2014). Patients’ baseline demographic, clinical and hospital-related characteristics are summarized in Table 2. The majority of patients were 45–64 years old (42.38%), female (73.54%), White (60.74%), covered by non-Medicare insurance (68.65%), admitted electively (90.05%), and had undergone total thyroidectomy (72.55%). Among the study cohort, 302 (0.79%) patients were determined to be frail, of which 50.99% were ≥ 65 years old, 41.39% male and 58.61% female. Significant differences were found between the frailty and non-frailty groups in age, gender, race, household income, insurance status, admission type, procedure type, whether or not required cervical lymph node dissection, metastasis to the lung, metastasis to the bone, hospital region and hospital volume (all *P* < 0.001) (Table 2).

**Table 2:** Demographic, clinical and hospital characteristics

	All patients (***n*** = 38,202)	Non-frailty (***n*** = 37,900)	Frailty (***n*** = 302)	***p***-value
**Age group**				< 0.0001
< 45	13,448 (35.20%)	13,400 (35.36%)	48 (15.89%)	
45–64	16,189 (42.38%)	16,089 (42.45%)	100 (33.11%)	
65+	8565 (22.42%)	8411 (22.19%)	154 (50.99%)	
**Gender**				< 0.0001
Female	27,898 (73.54%)	27,721 (73.66%)	177 (58.61%)	
Male	10,036 (26.46%)	9911 (26.34%)	125 (41.39%)	
Missing	268			
**Race**				< 0.0001
White	23,203 (60.74%)	23,023 (60.75%)	180 (59.60%)	
Black	2261 (5.92%)	2229 (5.88%)	32 (10.60%)	
Hispanic	3919 (10.26%)	3878 (10.23%)	41 (13.58%)	
Asian/Pacific Islander	1920 (5.03%)	1914 (5.05%)	6 (1.99%)	
Others	6899 (18.06%)	6856 (18.09%)	43 (14.24%)	
**Household income**				< 0.0001
0-25th percentile	6985 (18.69%)	6897 (18.61%)	88 (29.73%)	
26th–50th percentile	8268 (22.13%)	8182 (22.07%)	86 (29.05%)	
51th–75th percentile	9358 (25.04%)	9298 (25.08%)	60 (20.27%)	
76th–100th percentile	12,754 (34.13%)	12,692 (34.24%)	62 (20.95%)	
Missing	837			
**Insurance status/Primary Payer**				< 0.0001
Medicare/Medicaid	11,963 (31.35%)	11,762 (31.07%)	201 (66.56%)	
Non-Medicare	26,196 (68.65%)	26,095 (68.93%)	101 (33.44%)	
Missing	43			
**Admission type**				< 0.0001
Elective (ref)	34,307 (90.05%)	34,106 (90.24%)	201 (66.78%)	
Emergent	3790 (9.95%)	3690 (9.76%)	100 (32.22%)	
**Procedure type**				0.001
Unilateral/partial thyroidectomy	9270 (24.27%)	9178 (24.22%)	92 (30.46%)	
Total (complete) thyroidectomy	27,715 (72.55%)	27,522 (72.62%)	193 (63.91%)	
Substernal	1217 (3.19%)	1200 (3.17%)	17 (5.63%)	
**Required cervical LN dissection**				< 0.0001
No (ref)	29,102 (76.18%)	28,918 (76.30%)	184 (60.93%)	
Yes	9100 (23.82%)	8982 (23.70%)	118 (39.07%)	
**Metastasis to the lungs**				< 0.0001
No (ref)	37,417 (97.95%)	37,171 (98.08%)	246 (81.46%)	
Yes	785 (2.05%)	729 (1.92%)	56 (18.54%)	
**Metastasis to the bone**				< 0.0001
No (ref)	37,984 (99.43%)	37,700 (99.47%)	284 (94.04%)	
Yes	218 (0.57%)	200 (0.53%)	18 (5.96%)	
**Smoking**				0.133
None (ref)	32,391 (84.79%)	32,134 (84.79%)	257 (85.10%)	
Former	3193 (8.36%)	3175 (8.38%)	18 (5.96%)	
Current	2618 (8.94%)	2591 (6.84%)	27 (8.94%)	
**Overweight and obesity**				0.198
No (ref)	34,484 (90.27%)	34,218 (90.28%)	266 (88.08%)	
Yes	3718 (9.73%)	3682 (9.72%)	36 (11.92%)	
CCI				–
0–1 (ref)	33,916 (88.78%)	33,722 (88.98%)	194 (64.24%)	
2	2927 (7.66%)	2864 (7.56%)	63 (20.86%)	
3+	1359 (3.56%)	1314 (3.47%)	45 (14.90%)	
**Hospital characteristics**				
Hospital bed size				0.348
Small (ref)	4245 (11.16%)	4220 (11.18%)	25 (8.50%)	
Medium	7228 (19.00%)	7170 (19.00%)	7228 (19.00%)	
Large	26,566 (69.84%)	26,355 (69.82%)	211 (71.77%)	
Missing	163			
Location/teaching status				0.326
Rural	1940 (5.10%)	1926 (5.10%)	14 (4.76%)	
Urban nonteaching	11,264 (29.61%)	11,188 (29.64%)	76 (25.85%)	
Urban teaching	24,835 (65.29%)	24,631 (65.26%)	204 (69.39%)	
Missing	163			
Hospital region				< 0.0001
Northeast (ref)	12,236 (32.03%)	12,184 (32.15%)	52 (17.22%)	
Midwest	6444 (16.87%)	6378 (16.83%)	66 (21.85%)	
South	9563 (23.03%)	9456 (24.95%)	107 (35.43%)	
West	9959 (26.07%)	9882 (26.07%)	77 (25.50%)	
Hospital volume (surgeries/year)				< 0.0001
Low (< 8)	12,042 (31.52%)	11,901 (31.40%)	141 (46.69%)	
Intermediate (8–44)	17,107 (44.78%)	16,983 (44.81%)	124 (41.06%)	
High (> 44)	9053 (23.70%)	9016 (23.79%)	37 (12.25%)	

The inpatient outcomes of the patients after thyroidectomy are shown in Table 3. Significant differences were found between frailty and non-frailty groups in all four outcomes of interest: in-hospital mortality, prolonged length of stay, any surgical complications and medical complications, with a greater proportion of these outcomes observed in frail patients (all *P* < 0.001) (Table 3).

**Table 3:** Postoperative outcomes

	All patients (***n*** = 38,202)	Non-frailty (***n*** = 37,900)	Frailty (***n*** = 302)	***p***-value
**In-hospital death**				< 0.0001
Alive	38,138 (99.83%)	37,847 (99.86%)	291 (96.36%)	
Dead	64 (0.17%)	53 (0.14%)	11 (3.64%)	
**Prolonged LOS**				< 0.0001
N (<75th percentile) (< 2 days)	22,932 (60.03%)	22,892 (60.40%)	40 (13.25%)	
Y (> = 75th percentile) (> = 2 days)	15,270 (39.97%)	15,008 (39.60%)	262 (86.75%)	
**Surgical complications**	5388 (14.10%)	5253 (13.86%)	135 (44.70%)	< 0.0001
Tracheostomy	38 (0.10%)	33 (0.09%)	5 (1.66%)	< 0.0001
Hoarseness	270 (0.71%)	260 (0.69%)	10 (3.31%)	< 0.0001
Hemorrhage, hematoma, or seroma	784 (2.05%)	734 (1.94%)	50 (16.56%)	< 0.0001
Hypocalcemia	3587 (9.39%)	3540 (9.34%)	47 (15.56%)	0.0007
Vocal cord paresis or paralysis	921 (2.41%)	866 (2.28%)	55 (18.21%)	< 0.0001
Wound/ Technical complications	453 (1.19%)	427 (1.13%)	26 (8.61%)	< 0.0001
**Medical complications**	2690 (7.04%)	2523 (6.66%)	167 (55.30%)	< 0.0001
Cardiovascular	1672 (4.38%)	1600 (4.22%)	72 (23.84%)	< 0.0001
Pulmonary	521 (1.36%)	450 (1.19%)	71 (23.51%)	< 0.0001
Acute kidney injury	190 (0.50%)	155 (0.41%)	35 (11.59%)	< 0.0001
Pneumonia	604 (1.58%)	515 (1.36%)	89 (29.47%)	< 0.0001
Infection/Sepsis	397 (1.04%)	336 (0.89%)	61 (20.20%)	< 0.0001

### Associations between inpatient outcomes and frailty

The results of univariate and multivariate regression analysis on associations between frailty and inpatient outcomes are summarized in Table 4. Univariate analysis revealed that frailty was significantly associated with increased odds of in-hospital mortality (OR: 26.993, 95% CI: 13.958–52.203), prolonged length of stay (OR: 9.986, 95% CI: 7.156–13.936), any surgical complications (OR: 5.027, 95% CI: 3.999–6.319) and medical complications (OR: 17.363, 95% CI: 13.790–21.863) as compared to non-frailty. After adjustments for age, gender, race, household income, insurance status, admission type, procedure type, whether or not required cervical LN dissection, metastasis to the lung, metastasis to the bone, smoking status, overweight and obesity status, Charlson Comorbidity Index, hospital characteristics and hospital volume of thyroidectomy cases, frailty remained significantly associated with higher odds of in-hospital mortality (OR: 3.839, 95% CI: 1.738–8.480), prolonged length of stay (OR: 5.420, 95% CI: 3.799–7.733), any surgical complication (OR: 3.144, 95% CI: 2.443–4.045) and medical complication (OR: 6.734, 95% CI: 5.099–8.893) as compared with non-frailty. (Table 4) The details of all variables in the regression analyses are shown in Supplementary Table 1 and 2.

**Table 4:** Associations between outcomes and frailty

	Frailty			
	OR	95% CI	aOR^a^	95% CI
In-hospital death	**26.993**	**13.958–52.203**	**3.839**	**1.738–8.480**
Prolonged LOS	**9.986**	**7.156–13.936**	**5.420**	**3.799–7.733**
Surgical complications	**5.027**	**3.999–6.319**	**3.144**	**2.443–4.045**
Medical complications	**17.363**	**13.790–21.863**	**6.734**	**5.099–8.893**

The significance value is shown in bold

^a^ Multivariate analysis was adjusted for age, gender, race, household income, insurance status/primary payer, admission type, procedure type, required cervical LN dissection, metastasis to the lungs, metastasis to the bone, smoking, overweight and obesity, CCI and hospital characteristics

### Associations between outcomes and frailty according to age and number of comorbidities

Stratified analysis between frailty and the outcomes of interest according to age and number of comorbidities are summarized in Table 5. Frailty was significantly associated with increased odds of all inpatient outcomes among both older (> = 65 years old) and younger (< 65 years old) subgroups except for in-hospital death. Among the older subgroup, the adjusted odds ratio for frailty was 4.099 (95% CI: 1.736–9.679) for in-hospital mortality, 6.164 (95% CI: 3.514–10.812) for prolonged length of stay, 3.736 (95% CI: 2.620–5.328) for surgical complications, and 5.970, 95% CI: 4.088–8.720 for medical complications. Among the younger subgroup, the adjusted odds ratios were 4.738 (95% CI: 2.987–7.516) for prolonged length of stay, 2.636 (95% CI: 1.824–3.807) for surgical complications and 7.735 (95% CI: 5.205–11.495) for medical complications.

**Table 5:** Analysis of associations between outcomes and frailty stratified by age and CCI

	Frailty			
	OR	95% CI	aOR	95% CI
**Age < 65 (*n*** **= 29,637)**^**a**^				
In-hospital death	**15.424**	**2.005–118.670**	3.835	0.356–41.250
Prolonged LOS	**8.340**	**5.384–12.920**	**4.738**	**2.987–7.516**
Surgical complications	**3.650**	**2.604–5.116**	**2.636**	**1.824–3.807**
Medical complications	**19.339**	**13.904–26.898**	**7.735**	**5.205–11.495**
**Age > =65 (*n*** **= 8565)**^**a**^				
In-hospital death	**14.534**	**7.130–29.628**	**4.099**	**1.736–9.679**
Prolonged LOS	**10.834**	**6.445–18.213**	**6.164**	**3.514–10.812**
Surgical complications	**5.993**	**4.344–8.268**	**3.736**	**2.620–5.328**
Medical complications	**10.037**	**7.151–14.086**	**5.970**	**4.088–8.720**
**CCI < 3 (*n*** **= 36,843)**^**b**^				
In-hospital death	**24.970**	**11.097–56.185**	**3.672**	**1.357–9.934**
Prolonged LOS	**9.041**	**6.403–12.767**	**5.207**	**3.599–7.533**
Surgical complications	**5.281**	**4.124–6.763**	**3.327**	**2.530–4.375**
Medical complications	**18.023**	**14.055–23.111**	**7.702**	**5.752–10.314**
**CCI > =3 (*n*** **= 1359)**^**b**^				
In-hospital death	**10.585**	**3.274–34.226**	4.106	0.781–21.593
Prolonged LOS	**15.300**	**3.696–63.339**	**10.952**	**2.579–46.507**
Surgical complications	**2.837**	**1.538–5.233**	**2.859**	**1.461–5.593**
Medical complications	**5.587**	**2.805–11.130**	**5.297**	**2.363–11.870**

The significance value is shown in bold

*CCI* Charlson Comorbidity Index

^a^ Multivariate analysis was adjusted for gender, race, household income, insurance status/primary payer, admission type, procedure type, required cervical LN dissection, metastasis to the lung, metastasis to the bong, smoking, overweight and obesity, CCI and hospital characteristics

^b^ Multivariate analysis was adjusted for age, gender, race, household income, insurance status/primary payer, admission type, procedure type, required cervical LN dissection, metastasis to the lungs, metastasis to the bone, smoking, overweight and obesity and hospital characteristics

For comorbidities, patients were stratified into higher CCI (> = 3) and lower CCI (< 3) subgroups. In the lower CCI subgroup, frailty was associated with increased odds of in-hospital mortality (aOR: 3.672, 95% CI: 1.357–9.934), prolonged length of stay (aOR: 5.207, 95% CI: 3.599–7.533), surgical complications (aOR: 3.327, 95% CI: 2.530–4.375) and medical complications (aOR: 7.702, 95% CI: 5.752–10.314). In the higher CCI subgroup, frail patients had significantly higher odds of prolonged length of stay (aOR: 10.952, 95% CI: 2.579–46.507), surgical complications (aOR: 2.859, 95% CI: 1.461–5.593) and medical complications (aOR: 5.297, 95% CI: 2.363–11.870) as compared to non-frail patients, except for mortality (Table 5).

## Discussion

Results of the present study have shown that, after adjusting for all relevant comorbidities and clinical characteristics, hospitalized adult patients with preoperative frailty who underwent surgery for thyroid cancer are at over 3 times risk of in-hospital death and having surgical complications, and at over 5 times risk of having medical complications and prolonged length of stay compared to patients without frailty. The adverse predictive role of on the inpatient outcomes remained significant across subgroups, including younger or older age and number of comorbidities.

The effects of frailty in patients receiving thyroidectomy have been investigated by other authors, reporting surgeries performed for benign thyroid conditions mostly [14–16]. Among patients with multimodal goiter who underwent thyroidectomy, the frailty index provided more reliable risk assessment than age for complications associated with thyroidectomy [14]. In the present study, frailty was independently associated with increased risk of having medical complications, surgical complications and prolonged length of stay among both older (> = 65 years old) and younger (< 65 years old) patients. In previous studies focused on head and neck cancer patients, frailty was shown to be a stronger predictor than age for prolonged length of stay, surgical complications or medical complications [6, 19]. It was suggested that preoperative frailty assessment can provide useful information about health status and predictive information about outcomes; in cancer patients, in particular, tolerance to chemotherapy and radiotherapy can be predicted independent of age [6]. Nieman et al. [19] found frailty to be an independent predictor of postoperative morbidity and mortality, length of hospital stays and related costs in patients undergoing surgery for head and neck cancer. In that study, interactions with comorbidities also had a greater impact on complications and length of stay when accompanied by frailty. The findings of these studies and ours together contribute to the expanding literature highlighting the relevance of frailty rather than that of chronological age in preoperative decision making and perioperative patient care.

A previous study documented that rehospitalization among elderly patients in Medicare beneficiaries with thyroid cancer after thyroidectomy is both prevalent and costly, thus further predictors should be studied to enhance preoperative risk stratification, improve discharge planning, and increase outpatient support [13]. The present study did not evaluate readmission because the data were not available in the NIS database, which collects admission data separately for prior or subsequent admissions. While the present study focused only on inpatient outcomes provided by the NIS database, it was not the main focus of the investigation.

Evidence indicates that frailty assessment using risk-stratification tools are of particular help in understanding risks associated with individual older adult patients undergoing emergent general surgery as well as to assist with postoperative management and improve geriatric-centered outcomes [23]. Preoperative risk indices for frailty are being used more and more in patient undergoing thyroid and parathyroid surgery to predict major adverse events, including death with 30 days of surgery [16]. The present study defined frail and non-frail groups based on 10 clusters of frailty-defining diagnoses that comprise the Johns Hopkins Adjusted Clinical Groups (ACG) frailty-defining diagnosis indicator, a binary variable that uses ICD-9 codes assigned during admission [18, 19]. Among multiple measures of frailty in common use, the Johns Hopkins ACG frailty-defining diagnoses indicator was developed and validated recently to be used specifically with health administrative data, and is not intended to distinguish between degrees of frailty. Briefly, alternatives include the frailty phenotype reported by Fried et al. [1], which is based on five criteria: unintentional weight loss, exhaustion, decreased grip strength, decreased walking speed and low physical activity. The Rockwood index calculates subjective deficits that provide useful predictive information about frailty [24]. The ACG indicator used in the present study has been increasingly applied in studies based on administrative databases, but this new tool is not yet as widely used as other established indices.

### Strengths and limitations

The main strength of the present study and its findings is the use of the NIS database, a large and comprehensive database that closely represents the population of the United States and allows results to be generalized to a national population. The first analysis focused on the effects of frailty on the postoperative outcomes of older adults undergoing surgery for thyroid malignancy. Important confounding variables such as patients’ comorbidities and hospital characteristics, including hospital volume, were considered and adjusted in the analyses.

Nevertheless, this study has a few limitations that may interfere with the analysis and interpretation of result. The ICD-9 coding system was used to identify comorbidities in the included patients. Although comorbidities were graded based on the Charlson Index, the severity of individual comorbidities were unknown, which has the possibility of skewing results. Also, Johns Hopkins ACG frailty-defining index relies on ICD-9 codes. Frailty may be underestimated due to under-coding of the frailty-defining diagnoses. In addition, the index does not allow for defining degrees of frailty. As noted in previous work with the NIS database, the database lacks information on adjuvant therapy, subtype, grade, and stage of thyroid cancer, which may possibly bias the results. The NIS database lacks patients’ follow-up data after discharge, precluding the evaluation of later morbidity and mortality. Certain other confounding variables not collected by the NIS such as operation time may complicate analysis and limit the interpretation of results. Further, other important outcomes such as readmission or quality of life are not available in the database, limiting our evaluation and comparison of patient data.

## Conclusions

Frailty is associated with an increased risk for adverse inpatient outcomes, including prolonged hospital stay, increased surgical and medical complications and mortality in patients undergoing surgery for thyroid malignancy. These associations are independent of age, comorbidities and hospital volume. The findings of this study may provide valuable information for preoperative risk stratification, which may help surgeons to counsel patients appropriately about the risks of surgery, and could ultimately lead to better treatment decisions and care plans for older adults undergoing surgery for thyroid malignancy.

## Supplementary information

**Additional file 1.**

## Data Availability

The datasets used during the current study are available from the corresponding author on reasonable request.

## Abbreviations

NIS: Nationwide Inpatient Sample
NIH: National Institutes of Health
HCUP: Healthcare Cost and Utilization Project
IRB: Institutional Review Board
ACG: Adjusted Clinical Groups

## References

[1] Fried LP, Tangen CM, Walston J, Newman AB, Hirsch C, Gottdiener J (2001). Frailty in older adults: evidence for a phenotype. J Gerontol A Biol Sci Med Sci.

[2] Makary MA, Segev DL, Pronovost PJ, Syin D, Bandeen-Roche K, Patel P (2010). Frailty as a predictor of surgical outcomes in older patients. J Am Coll Surg.

[3] Czobor NR, Lehot JJ, Holndonner-Kirst E, Tully PJ, Gal J, Szekely A (2019). Frailty in patients undergoing vascular surgery: a narrative review of current evidence. Ther Clin Risk Manag.

[4] Kim DH, Kim CA, Placide S, Lipsitz LA, Marcantonio ER (2016). Preoperative frailty assessment and outcomes at 6 months or later in older adults undergoing cardiac surgical procedures: a systematic review. Ann Intern Med.

[5] McIsaac DI, Bryson GL, van Walraven C (2016). Association of Frailty and 1-year postoperative mortality following major elective noncardiac surgery: a population-based cohort study. JAMA Surg.

[6] Noor A, Gibb C, Boase S, Hodge JC, Krishnan S, Foreman A (2018). Frailty in geriatric head and neck cancer: a contemporary review. Laryngoscope.

[7] Handforth C, Clegg A, Young C, Simpkins S, Seymour MT, Selby PJ (2015). The prevalence and outcomes of frailty in older cancer patients: a systematic review. Ann Oncol.

[8] Surveillance, Epidemiology and End Results (SEER) data, http://seer.cancer.gov/statefacts/htm/thyro.html SEER (Accessed 20 Feb 2020).

[9] Pellegriti G, Frasca F, Regalbuto C, Squatrito S, Vigneri R. Worldwide increasing incidence of thyroid cancer: update on epidemiology and risk factors. J Cancer Epidemiol. 2013. 10.1155/2013/965212. 10.1155/2013/965212 PMC3664492 23737785

[10] Davies L, Welch HG (2014). Current thyroid cancer trends in the United States. JAMA Otolaryngol Head Neck Surg.

[11] Joseph KR, Edirimanne S, Eslick GD (2019). Thyroidectomy for thyroid cancer in the elderly: a meta-analysis. Eur J Surg Oncol.

[12] Boltz MM, Hollenbeak CS, Schaefer E, Goldenberg D, Saunders BD (2013). Attributable costs of differentiated thyroid cancer in the elderly Medicare population. Surgery.

[13] Tuggle CT, Park LS, Roman S, Udelsman R, Sosa JA (2010). Rehospitalization among elderly patients with thyroid cancer after thyroidectomy are prevalent and costly. Ann Surg Oncol.

[14] Finnerty BM, Gray KD, Ullmann TM, Zarnegar R, Fahey TJ 3rd, Beninato T. Frailty is more predictive than age for complications after thyroidectomy for multinodular goiter. World J Surg. 2020. 10.1007/s00268-020-05422-4. 10.1007/s00268-020-05422-4 32052107

[15] Seib CD, Rochefort H, Chomsky-Higgins K, Gosnell JE, Suh I, Shen WT (2018). Association of Patient Frailty with increased morbidity after common ambulatory general surgery operations. JAMA Surg.

[16] Mascarella MA, Milad D, Richardson K, Mlynarek A, Payne RJ, Forest VI, et al. Preoperative risk index among patients undergoing thyroid or parathyroid surgery. JAMA Otolaryngol Head Neck Surg. 2019. 10.1001/jamaoto.2019.2413. 10.1001/jamaoto.2019.2413 PMC6735422 31486838

[17] NIS Description of Data Elements (2017). Healthcare cost and utilization project (HCUP).

[18] The Johns Hopkins University (2015). ACG system: version 11.0 technical reference guide.

[19] Nieman CL, Pitman KT, Tufaro AP, Eisele DW, Frick KD, Gourin CG (2018). The effect of frailty on short-term outcomes after head and neck cancer surgery. Laryngoscope.

[20] Charlson ME, Pompei P, Ales KL, MacKenzie CR (1987). A new method of classifying prognostic comorbidity in longitudinal studies: development and validation. J Chronic Dis.

[21] Romano PS, Roos LL, Jollis JG (1993). Presentation adapting a clinical comorbidity index for use with ICD-9-CM administrative data: differing perspectives. J Clin Epidemiol.

[22] Gooi Z, Gourin CG, Boahene KD, Byrne PJ, Richmon JD (2015). Temporal trends in head and neck cancer surgery reconstruction. Head Neck.

[23] Ko FC (2019). Preoperative frailty evaluation: a promising risk-stratification tool in older adults undergoing general surgery. Clin Ther.

[24] Rockwood K, Song X, MacKnight C, Bergman H, Hogan DB, McDowell I (2005). A global clinical measure of fitness and frailty in elderly people. CMAJ.

