# Supplementary material for: Impact of frailty on inpatient outcomes in thyroid cancer surgery: 10-year results from the U.S. national inpatient sample
Source: J Otolaryngol Head Neck Surg. 2020 Jul 22;49:51. doi: 10.1186/s40463-020-00450-5 (PMC7376848; doi:10.1186/s40463-020-00450-5)
Supplement: Supplementary file 1 — Additional file 1. [file 40463_2020_450_MOESM1_ESM.docx]

| **Supplementary Table 1. Univariate analysis of associations between outcomes and all variables.** | | | | | | | | |
| --- | --- | --- | --- | --- | --- | --- | --- | --- |
|  | **In-hospital**  **death** | | **Prolonged LOS**  **(of survival)** | | **Surgical**  **complications** | | **Medical**  **complications** | |
|  | **OR** | **95% CI** | **OR** | **95% CI** | **OR** | **95% CI** | **OR** | **95% CI** |
| **Frailty** |  |  |  |  |  |  |  |  |
| No | Ref | Ref | Ref | Ref | Ref | Ref | Ref | Ref |
| Yes | **26.993** | **13.958-52.203** | **9.986** | **7.156-13.936** | **5.027** | **3.999-6.319** | **17.363** | **13.790-21.863** |
| **Age** |  |  |  |  |  |  |  |  |
| <45 | Ref | Ref | Ref | Ref | Ref | Ref | Ref | Ref |
| 45-64 | **4.987** | **1.116-22.286** | **0.953** | **0.909-0.998** | **0.902** | **0.844-0.965** | **2.876** | **2.511-3.294** |
| 65+ | **39.477** | **9.603-162.282** | **1.281** | **1.213-1.353** | **1.224** | **1.135-1.319** | **9.800** | **8.597-11.171** |
| **Gender** |  |  |  |  |  |  |  |  |
| Female | Ref | Ref | Ref | Ref | Ref | Ref | Ref | Ref |
| Male | **3.158** | **1.932-5.162** | **1.255** | **1.198-1.314** | 1.061 | 0.995-1.132 | **1.912** | **1.763-2.073** |
| **Race** |  |  |  |  |  |  |  |  |
| White | Ref | Ref | Ref | Ref | Ref | Ref | Ref | Ref |
| Black | 1.754 | 0.786-3.915 | **1.467** | **1.345-1.600** | 1.061 | 0.937-1.202 | **1.514** | **1.313-1.744** |
| Hispanic | 0.288 | 0.070-1.193 | **1.625** | **1.518-1.739** | **1.455** | **1.331-1.591** | **0.751** | **0.650-0.868** |
| Asian/Pacific Islander | 1.179 | 0.422-3.296 | **1.318** | **1.200-1.448** | **1.210** | **1.064-1.376** | **0.669** | **0.542-0.825** |
| Others | 0.820 | 0.411-1.638 | **1.208** | **1.144-1.276** | 1.036 | 0.958-1.120 | **0.813** | **0.728-0.908** |
| **Household Income** |  |  |  |  |  |  |  |  |
| 0-25th | Ref | Ref | Ref | Ref | Ref | Ref | Ref | Ref |
| 26th-50th | 0.619 | 0.284-1.349 | **0.851** | **0.978-0.907** | 0.983 | 0.901-1.072 | 0.933 | 0.832-1.045 |
| 51th-75th | 0.796 | 0.393-1.611 | **0.746** | **0.701-0.794** | **0.871** | **0.798-0.949** | **0.750** | **0.668-0.842** |
| 76th-100th | 0.657 | 0.331-1.304 | **0.564** | **0.531-0.599** | **0.724** | **0.667-0.787** | **0.602** | **0.538-0.674** |
| **Insurance status /primary payer** |  |  |  |  |  |  |  |  |
| Medicare/Medicaid | Ref | Ref | Ref | Ref | Ref | Ref | Ref | Ref |
| Non-Medicare | **0.127** | **0.070-0.231** | **0.692** | **0.662-0.723** | **0.805** | **0.758-0.856** | **0.235** | **0.216-0.255** |
| **Admission type** |  |  |  |  |  |  |  |  |
| Elective | Ref | Ref | Ref | Ref | Ref | Ref | Ref | Ref |
| Emergent | **9.712** | **5.941-15.874** | **1.762** | **1.647-1.885** | **1.371** | **1.255-1.498** | **2.210** | **1.992-2.452** |
| **Procedure type** |  |  |  |  |  |  |  |  |
| Unilateral/partial thyroidectomy | Ref | Ref | Ref | Ref | Ref | Ref | Ref | Ref |
| Total (complete) thyroidectomy | **0.345** | **0.207-0.576** | **1.609** | **1.531-1.692** | **1.738** | **1.610-1.876** | **0.907** | **0.828-0.993** |
| Substernal | 1.315 | 0.508-3.402 | **2.235** | **1.981-2.521** | **2.282** | **1.947-2.673** | **1.619** | **1.334-1.963** |
| **Required cervical LN dissection** |  |  |  |  |  |  |  |  |
| No | Ref | Ref | Ref | Ref | Ref | Ref | Ref | Ref |
| Yes | 1.563 | 0.927-2.635 | **3.172** | **3.021-3.330** | **2.099** | **1.974-2.231** | **1.298** | **1.190-1.417** |
| **Metastasis to the lungs** |  |  |  |  |  |  |  |  |
| No | Ref | Ref | Ref | Ref | Ref | Ref | Ref | Ref |
| Yes | **43.686** | **26.598-71.753** | **6.701** | **5.597-8.023** | **4.250** | **3.673-4.917** | **7.131** | **6.112-8.320** |
| **Metastasis to the bone** |  |  |  |  |  |  |  |  |
| No | Ref | Ref | Ref | Ref | Ref | Ref | Ref | Ref |
| Yes | **8.675** | **2.701-27.861** | **3.336** | **2.503-4.446** | **1.634** | **1.179-2.265** | **5.596** | **4.170-7.510** |
| **Smoking** |  |  |  |  |  |  |  |  |
| None | Ref | Ref | Ref | Ref | Ref | Ref | Ref | Ref |
| Former | 0.338 | 0.083-1.382 | 1.047 | 0.972-1.127 | 1.078 | 0.973-1.194 | **1.240** | **1.086-1.416** |
| Current | 0.412 | 0.101-1.686 | **1.128** | **1.040-1.222** | **1.208** | **1.084-1.346** | 1.142 | 0.984-1.327 |
| **Overweight and Obesity** |  |  |  |  |  |  |  |  |
| No | Ref | Ref | Ref | Ref | Ref | Ref | Ref | Ref |
| Yes | 1.519 | 0.750-3.076 | **1.325** | **1.238-1.418** | **1.289** | **1.177-1.411** | **2.109** | **1.898-2.345** |
| **CCI** |  |  |  |  |  |  |  |  |
| 0-1 | Ref | Ref | Ref | Ref | Ref | Ref | Ref | Ref |
| 2 | **3.056** | **1.521-6.140** | **1.392** | **1.290-1.501** | **1.341** | **1.213-1.483** | **4.202** | **3.776-4.675** |
| 3+ | **10.621** | **5.907-19.097** | **2.346** | **2.101-2.620** | **1.562** | **1.361-1.791** | **11.339** | **10.050-12.793** |
| **Hospital characteristics** |  |  |  |  |  |  |  |  |
| Hospital bed size |  |  |  |  |  |  |  |  |
| Small | Ref | Ref | Ref | Ref | Ref | Ref | Ref | Ref |
| Medium | **8.236** | **1.083-62.655** | 1.028 | 0.951-1.110 | 1.020 | 0.916-1.137 | **1.274** | **1.089-1.490** |
| Large | **7.842** | **1.083-56.807** | 1.009 | 0.945-1.078 | 0.982 | 0.895-1.078 | **1.259** | **1.097-1.444** |
| Location/teaching status |  |  |  |  |  |  |  |  |
| Rural | Ref | Ref | Ref | Ref | Ref | Ref | Ref | Ref |
| Urban nonteaching | 0.550 | 0.201-1.504 | 1.074 | 0.974-1.184 | 0.957 | 0.834-1.099 | 0.954 | 0.793-1.147 |
| Urban teaching | 0.671 | 0.266-1.696 | **0.840** | **0.765-0.922** | 0.985 | 0.863-1.123 | 0.925 | 0.775-1.104 |
| Hospital region |  |  |  |  |  |  |  |  |
| Northeast | Ref | Ref | Ref | Ref | Ref | Ref | Ref | Ref |
| Midwest | 1.229 | 0.575-2.625 | **1.946** | **1.827-2.072** | **2.197** | **2.011-2.400** | **1.831** | **1.628-2.059** |
| South | **1.960** | **1.063-3.613** | **2.472** | **2.337-2.614** | **2.187** | **2.018-2.371** | **1.848** | **1.661-2.055** |
| West | 0.722 | 0.331-1.578 | **1.816** | **1.717-1.920** | **1.618** | **1.488-1.759** | **1.238** | **1.104-1.387** |
| Hospital volume (surgeries/year) |  |  |  |  |  |  |  |  |
| Low (<8) | Ref | Ref | Ref | Ref | Ref | Ref | Ref | Ref |
| Intermediate (8-44) | 0.578 | 0.333-1.003 | **0.771** | **0.735-0.808** | **0.737** | **0.691-0.785** | **0.731** | **0.671-0.797** |
| High (>44) | 0.617 | 0.319-1.192 | **0.495** | **0.467-0.524** | **0.488** | **0.449-0.531** | **0.497** | **0.443-0.558** |

| **Supplementary Table 2. Multivariate analysis of associations between outcomes and all variables.** | | | | | | | | |
| --- | --- | --- | --- | --- | --- | --- | --- | --- |
|  | **In-hospital**  **death** | | **Prolonged LOS**  **(of survival)** | | **Surgical**  **complications** | | **Medical**  **complications** | |
|  | **OR** | **95% CI** | **OR** | **95% CI** | **OR** | **95% CI** | **OR** | **95% CI** |
| **Frailty** |  |  |  |  |  |  |  |  |
| No | Ref | Ref | Ref | Ref | Ref | Ref | Ref | Ref |
| Yes | **3.839** | **1.738-8.480** | **5.420** | **3.799-7.733** | **3.144** | **2.443-4.045** | **6.734** | **5.099-8.893** |
| **Age** |  |  |  |  |  |  |  |  |
| <45 | Ref | Ref | Ref | Ref | Ref | Ref | Ref | Ref |
| 45-64 | **3.137** | **0.686-14.340** | **1.011** | **0.959-1.065** | **0.934** | **0.870-1.002** | **2.376** | **2.057-2.743** |
| 65+ | **10.991** | **2.353-51.341** | **1.003** | **0.927-1.084** | **1.148** | **1.035-1.273** | **4.631** | **3.940-5.443** |
| **Gender** |  |  |  |  |  |  |  |  |
| Female | Ref | Ref | Ref | Ref | Ref | Ref | Ref | Ref |
| Male | **2.003** | **1.142-3.512** | **1.055** | **1.001-1.111** | 0.907 | 0.845-0.973 | **1.450** | **1.320-1.592** |
| **Race** |  |  |  |  |  |  |  |  |
| White | Ref | Ref | Ref | Ref | Ref | Ref | Ref | Ref |
| Black | 1.446 | 0.553-3.779 | **1.379** | **1.250-1.520** | 0.946 | 0.826-1.083 | **1.125** | **0.949-1.334** |
| Hispanic | 0.420 | 0.096-1.834 | **1.327** | **1.228-1.434** | **1.291** | **1.170-1.425** | **0.787** | **0.666-0.932** |
| Asian/Pacific Islander | 2.536 | 0.810-7.942 | **1.466** | **1.321-1.626** | **1.358** | **1.186-1.557** | **0.939** | **0.746-1.184** |
| Others | 1.037 | 0.480-2.239 | **1.133** | **1.063-1.207** | 0.926 | 0.850-1.009 | **0.879** | **0.773-1.000** |
| **Household Income** |  |  |  |  |  |  |  |  |
| 0-25th | Ref | Ref | Ref | Ref | Ref | Ref | Ref | Ref |
| 26th-50th | 0.861 | 0.367-2.016 | **0.936** | **0.872-1.004** | 1.051 | 0.958-1.153 | 1.099 | 0.964-1.252 |
| 51th-75th | 1.513 | 0.683-3.350 | **0.852** | **0.794-0.914** | **0.979** | **0.892-1.074** | **0.999** | **0.874-1.142** |
| 76th-100th | 1.408 | 0.624-3.177 | **0.741** | **0.692-0.794** | **0.941** | **0.857-1.033** | **0.972** | **0.849-1.112** |
| **Insurance status /primary payer** |  |  |  |  |  |  |  |  |
| Medicare/Medicaid | Ref | Ref | Ref | Ref | Ref | Ref | Ref | Ref |
| Non-Medicare | **0.742** | **0.322-1.708** | **0.780** | **0.730-0.832** | **1.006** | **0.922-1.098** | **0.583** | **0.517-0.658** |
| **Admission type** |  |  |  |  |  |  |  |  |
| Elective | Ref | Ref | Ref | Ref | Ref | Ref | Ref | Ref |
| Emergent | **5.413** | **3.075-9.527** | **1.557** | **1.444-1.678** | **1.200** | **1.090-1.321** | **1.791** | **1.582-2.026** |
| **Procedure type** |  |  |  |  |  |  |  |  |
| Unilateral/partial thyroidectomy | Ref | Ref | Ref | Ref | Ref | Ref | Ref | Ref |
| Total (complete) thyroidectomy | **0.470** | **0.262-0.843** | **1.480** | **1.401-1.564** | **1.625** | **1.499-1.763** | **1.017** | **0.917-1.129** |
| Substernal | 0.686 | 0.213-2.207 | **1.744** | **1.528-1.989** | **1.808** | **1.530-2.138** | **1.233** | **0.985-1.543** |
| **Required cervical LN dissection** |  |  |  |  |  |  |  |  |
| No | Ref | Ref | Ref | Ref | Ref | Ref | Ref | Ref |
| Yes | 1.600 | 0.861-2.974 | **3.253** | **3.082-3.434** | **1.872** | **1.750-2.002** | **1.413** | **1.273-1.568** |
| **Metastasis to the lungs** |  |  |  |  |  |  |  |  |
| No | Ref | Ref | Ref | Ref | Ref | Ref | Ref | Ref |
| Yes | **18.131** | **9.827-33.452** | **4.322** | **3.553-5.257** | **3.013** | **2564-3.540** | **3.687** | **3.059-4.444** |
| **Metastasis to the bone** |  |  |  |  |  |  |  |  |
| No | Ref | Ref | Ref | Ref | Ref | Ref | Ref | Ref |
| Yes | **0.491** | **0.132-1.827** | **1.770** | **1.271-2.464** | **0.769** | **0.531-1.113** | **1.685** | **1.161-2.446** |
| **Smoking** |  |  |  |  |  |  |  |  |
| None | Ref | Ref | Ref | Ref | Ref | Ref | Ref | Ref |
| Former | 0.095 | 0.012-0.732 | 0.988 | 0.910-1.073 | 1.033 | 0.927-1.152 | **0.834** | **0.718-0.968** |
| Current | 0.625 | 0.149-2.632 | **1.006** | **0.921-1.100** | **1.135** | **1.011-1.273** | 1.057 | 0.892-1.252 |
| **Overweight and Obesity** |  |  |  |  |  |  |  |  |
| No | Ref | Ref | Ref | Ref | Ref | Ref | Ref | Ref |
| Yes | 1.152 | 0.497-2.668 | **1.187** | **1.101-1.280** | **1.167** | **1.060-1.285** | **1.850** | **1.637-2.092** |
| **CCI** |  |  |  |  |  |  |  |  |
| 0-1 | Ref | Ref | Ref | Ref | Ref | Ref | Ref | Ref |
| 2 | **1.668** | **0.747-3.724** | **1.408** | **1.295-1.530** | **1.318** | **1.184-1.467** | **3.022** | **2.682-3.404** |
| 3+ | **4.814** | **2.375-9.758** | **2.118** | **1.875-2.393** | **1.377** | **1.187-1.599** | **6.301** | **5.498-7.223** |
| **Hospital characteristics** |  |  |  |  |  |  |  |  |
| Hospital bed size |  |  |  |  |  |  |  |  |
| Small | Ref | Ref | Ref | Ref | Ref | Ref | Ref | Ref |
| Medium | **6.751** | **0.842-54.160** | 0.942 | 0.865-1.025 | 0.978 | 0.873-1.096 | **1.135** | **0.955-1.349** |
| Large | **6.289** | **0.841-47.035** | 1.030 | 0.956-1.109 | 1.085 | 0.983-1.198 | **1.257** | **1.079-1.465** |
| Location/teaching status |  |  |  |  |  |  |  |  |
| Rural | Ref | Ref | Ref | Ref | Ref | Ref | Ref | Ref |
| Urban nonteaching | 0.527 | 0.173-1.608 | 1.259 | 1.129-1.405 | 1.076 | 0.928-1.248 | 1.224 | 0.992-1.509 |
| Urban teaching | 0.651 | 0.220-1.924 | **1.017** | **0.913-1.134** | 1.269 | 1.097-1.469 | 1.392 | 1.132-1.713 |
| Hospital region |  |  |  |  |  |  |  |  |
| Northeast | Ref | Ref | Ref | Ref | Ref | Ref | Ref | Ref |
| Midwest | 0.956 | 0.371-2.459 | **1.550** | **1.438-1.670** | **1.849** | **1.671-2.046** | **1.367** | **1.183-1.579** |
| South | **1.713** | **0.791-3.706** | **1.896** | **1.777-2.023** | **1.779** | **1.626-1.947** | **1.334** | **1.174-1.515** |
| West | 0.742 | 0.297-1.859 | **1.456** | **1.364-1.555** | **1.399** | **1.275-1.537** | **1.156** | **1.009-1.324** |
| Hospital volume (surgeries/year) |  |  |  |  |  |  |  |  |
| Low (<8) | Ref | Ref | Ref | Ref | Ref | Ref | Ref | Ref |
| Intermediate (8-44) | 0.757 | 0.392-1.463 | **0.861** | **0.815-0.909** | **0.740** | **0.689-0.795** | **0.829** | **0.747-0.919** |
| High (>44) | 1.337 | 0.551-3.245 | **0.684** | **0.634-0.737** | **0.559** | **0.504-0.619** | **0.684** | **0.589-0.794** |
